# Age at First Delivery and Osteoporosis Risk in Korean Postmenopausal Women: The 2008–2011 Korea National Health and Nutrition Examination Survey (KNHANES)

**DOI:** 10.1371/journal.pone.0123665

**Published:** 2015-05-06

**Authors:** Bo Hyon Yun, Yun Rak Choi, Young Sik Choi, SiHyun Cho, Byung Seok Lee, Seok Kyo Seo

**Affiliations:** 1 Department of Obstetrics and Gynecology, Yonsei University College of Medicine, Seoul, Republic of Korea; 2 Institute of Women’s Life Medical Science, Yonsei University College of Medicine, Seoul, Republic of Korea; 3 Department of Orthopaedic Surgery, Yonsei University College of Medicine, Seoul, Republic of Korea; 4 Department of Obstetrics and Gynecology, Gangnam Severance Hospital, Yonsei University College of Medicine, Seoul, Republic of Korea; Innsbruck Medical University, AUSTRIA

## Abstract

It has been reported in several studies that there may be a significant correlation between reproductive history and the risk of osteoporosis due to the effect of estrogen. Under this hypothesis, however, it is unclear whether the age at first delivery has any major influences on the risk of osteoporosis. Therefore, this study aimed to investigate the relationship between the age at first delivery and the risk of osteoporosis in Korean menopausal women. This study was performed using data from the 2008–2011 Korea National Health and Nutrition Examination Survey and included 2,530 Korean postmenopausal women. The diagnosis of osteoporosis was made using the World Health Organization T-score criteria (T-score ≤ -2.5, at the femoral neck or lumbar spine). Participants were categorized into 3 groups according to age at first delivery: ≤23, 24–29, and ≥30 years. Older age, lower body mass index, lower calcium intake, later menarche, and earlier menopause increased the risk of osteoporosis, whereas hormone therapy and oral contraceptive use were associated with a decreased risk of osteoporosis. Postmenopausal women whose first delivery occurred at age 24–29 years were shown to have a significantly increased risk of osteoporosis (odds ratio, 2.124; 95% confidence interval, 1.096–4.113; P = 0.026) compared to those who first gave birth after the age of 30 years. These findings suggest that postmenopausal women whose first delivery occurred in their mid to late 20s, a period during which bone mass slowly accumulates to the peak, are at an increased risk of osteoporosis.

## Introduction

Osteoporosis is a major cause of morbidity, as osteoporosis-related fracture can result in disability and even death [1,2]. The risk of fracture depends on the peak bone mass and subsequent rate of bone loss. In women, peak bone mass is influenced by hereditary and endocrine factors, and bone loss is affected by genetic susceptibility, life style such as smoking, high alcohol consumption, and depletion of estrogen [3,4]. In postmenopausal women, there is a rapid decrease in bone mineral density due to estrogen depletion, resulting in a higher fracture risk and increased morbidity compared to similarly aged men [3,5].

Although bones in women are greatly affected by estrogen (either quantity or quality), results on the correlation between reproductive history and risk of osteoporosis due to the effects of estrogen are conflicting. Reproductive factors such as late menarche and parity have been suggested to be risk factors for postmenopausal osteoporosis, although this is controversial [6–10]. It is unclear whether age at the time of first pregnancy and effects of pregnancy on bone loss have major influences on the risk of osteoporosis. It is believed that reversible bone loss is likely to occur in most women during pregnancy, although osteoporosis associated with pregnancy is rare [11]. However, the impact of age at first delivery has shown contradictory results in studies. Adolescent pregnancy has been suggested to be a risk factor for postmenopausal osteoporosis [12], and women whose first pregnancy occurred after PBM acquisition (27 years of age in the study) with breast feeding history showed decreased prevalence of osteoporosis [13]. Nevertheless, another study has suggested that adolescent pregnancy seems to exert no significant influence on either acquisition of peak bone mass or future risk of osteoporosis [9].

In this study, we focused on possible reproductive factors that might affect bone mass formation [7,14] and bone loss, thereby aiming to investigate the relationship between reproductive factors and osteoporosis in Korean postmenopausal women and to evaluate risk factors for the development of osteoporosis.

## Materials and Methods

### Study population

This study was designed as a cross-sectional study based on data from the Korea National Health and Nutrition Examination Survey (KNHANES) from 2008 to 2011, including data from the KNHANES IV survey (data from 2008 and 2009) and the KNHANES V survey (data from 2010 and 2011). The KNHANES is a nationwide survey, conducted annually by the Korean Ministry of Health and Welfare since 1998, to investigate the health and nutritional status of the Korean people. KNHANES IV and V were each conducted for 3 years (2007–2009 and 2010–2012, respectively) using a stratified, multi-stage, clustered probability sampling method to select a representative sample of the non-institutionalized, civilian Korean population. Sampling units were randomly selected; in 2008, there were 200 randomly selected sampling units with 23 households in each unit (yielding 4,600 households), and in 2009–2011, there were 192 randomly selected sampling units with 20 households in each unit (yielding 3,840 households). For each year, the sampling units were newly selected and did not overlap with previous samples. The survey consisted of 3 parts: the Health Interview Survey, Nutrition Survey, and Health Examination Survey. Data were collected by household interviews and direct standardized physical examinations conducted in mobile examination centers. The interviewers were not provided with any information regarding specific participants before conducting the interviews, and all participants provided written consent forms prior to enrollment. The study was approved by the Yonsei University Health System, Severance Hospital, Institutional Review Board (4-2013-0322).

Among those who participated in the survey, there were 5,229 women aged 33–95 years who had not menstruated for at least 1 year. Postmenopausal women were defined as women over 45 years of age who answered that they had experienced menopause in the survey (n = 5017). To avoid selective bias, women aged 71–95 years were excluded (n = 840), since there is a higher prevalence of osteoporosis in the elderly. Women who had undergone hysterectomy (n = 431), which is known to increase the risk of early menopause [15,16], or bilateral oophorectomy (n = 170) were excluded. In addition, women were excluded from the study if they had a chronic condition that may have influenced bone metabolism, such as end-stage renal disease (n = 41), thyroid disease (n = 468), and cancer (n = 367), or if information regarding bone mineral density (BMD) or T-score of the femur neck or lumbar spine was not available (n = 170). Finally, 2,530 postmenopausal women were enrolled in the present study.

### Measurements

All participants underwent thorough physical examinations. Age, body weight, height, smoking and drinking history, exercise, and food and nutritional intake were recorded. Age was expressed in years. Current smokers were defined as those who smoked at least 1 cigarette daily during the previous 12 months. Drinking history was divided into 2 categories, depending on whether the participant had a history of drinking within the previous 12 months. Based on the International Physical Activity Questionnaire short-form scoring protocol [17], physical activity levels were divided into 3 categories: low, moderate, and high. The 24-h dietary recall method was used to collect data on food items consumed by participants. Energy and calcium intake were calculated based on the consumption of each food item. Information on reproductive factors, including age at menarche and menopause, age at first delivery, total number of pregnancies, breastfeeding history, use of oral contraceptives (OCs), and use of hormone therapy (HT), was collected. History of using OC or HT was defined as use of either of these medications for at least 1 month. Participants were divided into 3 groups based on the age of first delivery: ≤23, 24–29, and ≥30 years. These age groups were determined based on bone mass distribution of the female population from 10 to 85 years of age, from the data of KNHANES 2008 to 2011 (S1 Table and S1 Fig). We also calculated the duration from menarche to first delivery and the time elapsed since the onset of menopause. For those with a history of breastfeeding, we determined the total duration of breastfeeding.

Blood samples were collected for biochemical analyses from all subjects during the survey. Blood samples were collected early in the morning after an overnight fast. Serum 25-hydroxyvitamin D levels were measured by electrochemiluminescence immunoassay using a COBAS autoanalyzer (Roche Diagnostics, West Sussex, UK; intra- and inter-assay coefficients of variation <8% and <10%, respectively). All clinical analyses were performed by the Neodin Medical Institute, a laboratory certified by the Korean Ministry of Health and Welfare.

Body weight, anthropometrics, and BMD were measured with participants wearing light clothing, without shoes or jewelry. Body mass index (BMI) was calculated using measured height and weight (kilograms per square meter of body height). The BMD was measured in central skeletal sites (femoral neck, lumbar spine L1–L4) using dual energy X-ray absorptiometry (DXA; DISCOVERY-W; HOLOGIC Inc., Santa Clara, CA). The precision of DXA has been previously reported in the KNHANES, and the DXA instruments were calibrated using the methods described in a previous report [18]. The KNHANES calibrations were used for appropriate comparisons among data. The T-score, which is the derivation of a participant’s BMD from the weight-adjusted average peak BMD of a healthy population, was used to analyze the BMD data. Diagnosis of osteoporosis was made using the World Health Organization T-score criteria; osteoporosis was defined as a T-score lower than -2.5 at either the femoral neck or lumbar spine.

### Statistical analysis

Data were examined for normality of distribution. Data are presented as the mean ± standard deviation for continuous variables, and numbers and percentages are reported for categorical variables. Baseline characteristics were compared between the osteoporosis group and the non-osteoporosis group using the Student *t*-test for normally distributed continuous variables, and the chi-square test was used for normally distributed categorical variables. Binary logistic regression analysis was performed to investigate which reproductive factors affected the development of postmenopausal osteoporosis. A logistic model was made on the basis of goodness-of-fit of the model and multi-collinearity of the factors. The factors included in the logistic model were selected by the univariate analysis of the data and previously established risk factors for osteoporosis. Variables were included in the model if they achieved a P value <0.10 in the univariate analysis. If the P value did not meet the criteria, but was a previously established risk factor, it was included in the model. P < 0.05 was considered statistically significant.

## Results

**Fig 1:**
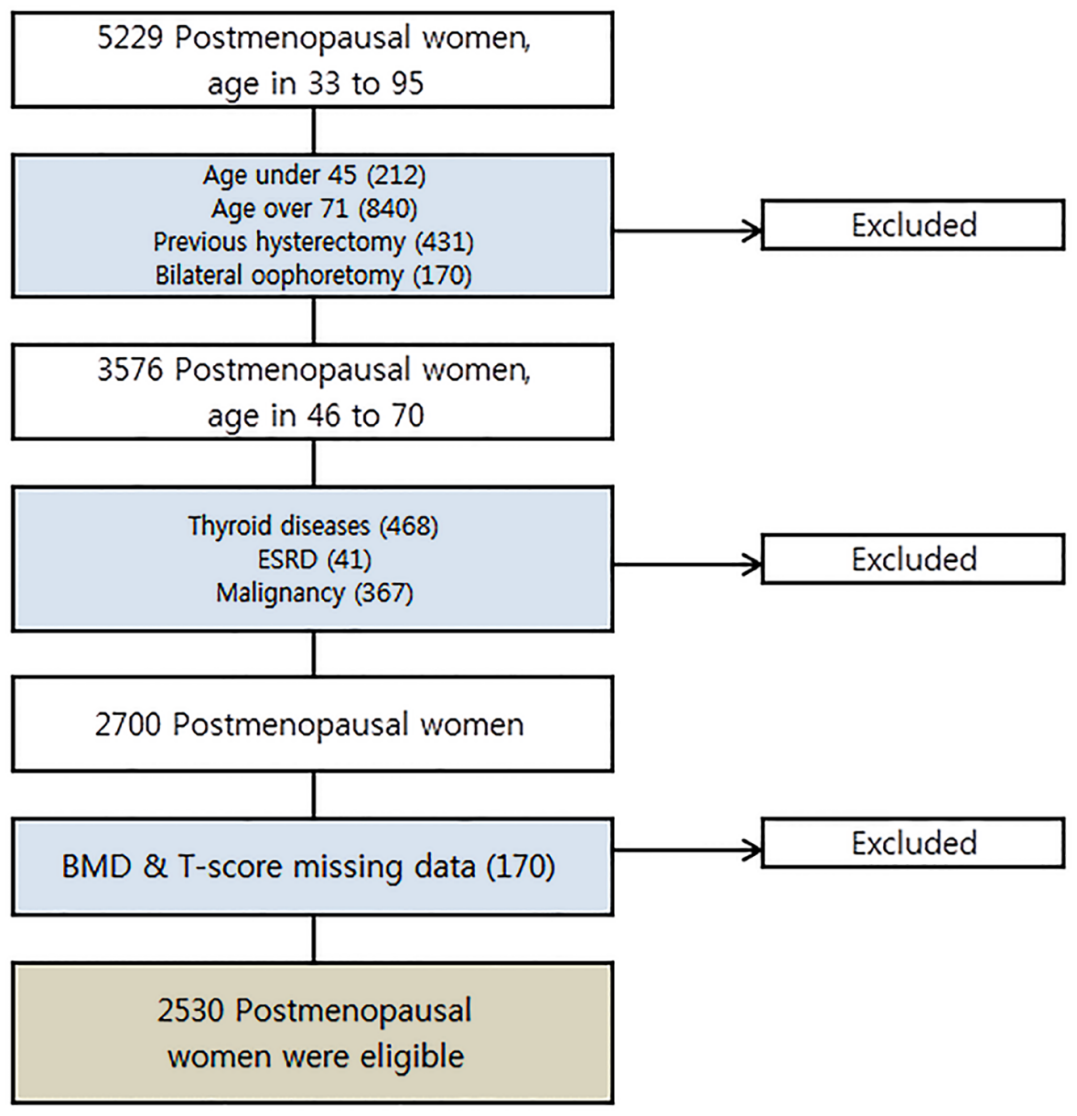
Flow chart of participant enrollment.

The flow chart of the study process is presented in Fig 1. The characteristics of the study population are shown in Table 1. As expected, there was a significant difference in mean BMD between the osteoporosis (femoral neck: 0.56 g/cm^2^; lumbar spine: 0.67 g/cm^2^) and non-osteoporosis groups (femoral neck: 0.68 g/cm^2^; lumbar spine: 0.88 g/cm^2^; P < 0.0001, respectively). In addition, the osteoporosis group exhibited a significantly higher age (62.88 vs. 58.58 years; P < 0.0001), lower BMI (23.61 vs. 24.47 kg/m^2^; P < 0.0001), and lower daily calcium intake (397.50 vs. 456.66 mg; P < 0.0001) than the non-osteoporosis group. Vitamin D deficiency was observed in both groups (18.52 vs. 18.84 ng/mL; P = 0.073); there were no significant differences in exercise and smoking history between the groups. In the osteoporosis group, the proportion of participants with osteoporosis at the femoral neck and lumbar spine was 50.19% and 95.11%, respectively; osteoporosis at both sites was observed in 30.43% of participants (data not shown).

**Table 1:** Baseline characteristics of study participants.

Variables		Osteoporosis (n = 673)	Non-osteoporosis (n = 1857)	P value
Age, years		62.88 ± 5.56	58.58 ± 5.95	<0.0001^1^
BMI, kg/m^2^		23.61 ± 2.99	24.47 ± 3.17	<0.0001
BMI, kg/m^2^	<25	480 (71.4%)	1097 (59.2%)	<0.0001
	≥25	192 (28.6%)	757 (40.8%)	
BMD (femoral neck, g/cm^2^)		0.56 ± 0.07	0.68 ± 0.89	<0.0001^1^
BMD (lumbar spine, g/cm^2^)		0.67 ± 0.08	0.88 ± 0.11	<0.0001^1^
T score (femoral neck)		-2.10 ± 0.80	-0.93 ± 0.90	<0.0001^1^
T score (lumbar spine)		-2.90 ± 0.63	-1.16 ± 0.91	<0.0001^1^
Serum Vitamin D [25(OH)D], ng/mL		18.52 ± 7.55	18.84 ± 6.92	0.073^1^
Ca intake, mg/day		397.50 ± 271.12	456.66 ± 320.33	<0.0001^1^
Exercise, days/week		2.18 ± 3.29	2.36 ± 3.23	0.102^1^
Smoking	Non-smoker	623 (93.0%)	1732 (93.5%)	0.887
	Past smoker	22 (3.3%)	59 (3.2%)	
	Current smoker	25 (3.7%)	62 (3.3%)	

^1^Mann—Whitney *U* test was performed for continuous variables that did not show normal distribution.

BMI, body mass index; BMD, bone mass density; Ca, calcium.

Data are presented as the mean ± standard deviation.

P values were obtained using the 2-sample *t*-test for continuous variables and the chi-square test for categorical variables.

The reproductive factors of the participants are presented in Table 2. The osteoporosis group had a significantly higher mean age at menarche than the non-osteoporosis group (16.20 vs. 15.70 years; P < 0.0001). There were no significant differences between the groups with regard to mean age at menopause (50.70 vs. 50.82 years; P = 0.389), gravidity (5.69 vs. 5.58; P = 0.792), and age at first delivery (23.96 vs. 24.15 years; P = 0.207). In addition, the osteoporosis group had a significantly lower incidence of oral contraceptive use (21.9% vs. 26.7%; P = 0.017) and HT use (12.7% vs. 21.7%; P < 0.0001).

**Table 2:** Reproductive characteristics of the study population.

Variables		Osteoporosis (n = 673)	Non-osteoporosis (n = 1857)	P value
Age at menarche, years		16.20 ± 2.01	15.70 ± 1.91	<0.0001
Age at menopause, years		50.70 ± 3.09	50.82 ± 2.87	0.128^1^
Gravidity, n		5.69 ± 8.78	5.58 ± 9.47	0.792
Time from menarche to first delivery, years		7.77 ± 3.85	8.49 ± 3.99	<0.0001
Time since menopause, years		12.07 ± 6.14	7.70 ± 5.88	<0.0001
Age at first delivery, years		23.96 ± 3.19	24.15 ± 3.33	0.207
First delivery age groups, n (%)	≤23 years	310 (46.5%)	821 (44.6%)	0.053
	24–29 years	331 (49.7%)	903 (49.1%)	
	≥30 years	25 (3.8%)	115 (6.3%)	
Time from menarche to first delivery, n (%)	≤5 years	199 (30.6%)	437 (24.2%)	0.021
	6–10 years	300 (46.1%)	860 (47.6%)	
	11–15 years	134 (20.6%)	430 (23.8%)	
	16–20 years	16 (2.5%)	70 (3.9%)	
	21–25 years	2 (0.3%)	8 (0.4%)	
	26–30 years	0 (0%)	1 (0%)	
History of breast feeding, n (%)	Yes	289 (95.1%)	838 (90.4%)	0.012
	No	15 (4.9%)	89 (9.6%)	
Total duration of breast feeding groups, n (%)	≤24 months	83 (28.8%)	368 (44.0%)	<0.0001
	25–48 months	94 (32.6%)	265(31.7%)	
	49–72 months	55 (19.1%)	112 (13.4%)	
	≥73 months	56 (19.4%)	91 (10.9%)	
History of OC use, n (%)	Yes	147 (21.9%)	494 (26.7%)	0.017
	No	523 (78.1%)	1358 (73.3%)	
History of HT use, n (%)	Yes	85 (12.7%)	401 (21.7%)	<0.0001
	No	585 (87.3%)	1447 (78.3%)	

^1^Mann—Whitney *U* test was performed for continuous variables that did not show normal distribution.

OC, oral contraceptive; HT, hormone therapy.

Data are presented as the mean ± standard deviation.

P values were obtained using the 2-sample *t*-test for continuous variables and the chi-square test for categorical variables.

A logistic model including the factors identified in the univariate analysis and previously known risk factors was then created. Incorporating potential influences of multiple factors on the development of postmenopausal osteoporosis, the results showed that the risk of postmenopausal osteoporosis increased significantly with a younger age at first delivery (Table 3). However, adjusting for possible risk factors, the influence of age at first delivery on osteoporosis was greater in those whose first delivery occurred between 24 and 29 years of age (odds ratio, 2.124; 95% confidence interval, 1.096–4.113; P = 0.026) than in those whose first delivery occurred at ≥30 years of age (Table 3). In addition, other previously reported risk factors for postmenopausal osteoporosis, including age, BMI, calcium intake, age at menarche and menopause, and HT and OC use, were confirmed to have a significant relationship with osteoporosis.

**Table 3:** Adjusted odds ratios of risk factors of postmenopausal osteoporosis.

Variables		Unadjusted OR (95% CI)	P value	Adjusted OR (95% CI)	P value
Age		1.132(1.114–1.151)	<0.0001	1.142(1.121–1.164)	<0.0001
BMI		1.725(1.425–2.089)	<0.0001	2.296(1.836–2.871)	<0.0001
Smoking status	Non-smoker	1		1	
	Past smoker	1.037(1.630–1.706)	0.887	1.261(0.715–2.222)	0.423
	Current smoker	1.121(0.698–1.800)	0.636	1.149(0.638–2.068)	0.644
Calcium intake		0.931(0.900–0.963)	<0.0001	0.959(0.924–0.995)	0.025
Serum Vitamin D		0.994(0.981–1.007)	0.332	0.990(0.976–1.004)	0.163
Exercise		0.983(0.956–1.011)	0.227	1.003(0.972–1.036)	0.844
Age at menarche		1.141(1.090–1.195)	<0.0001	1.110(1.020–1.207)	0.015
Age at menopause		0.986(0.956–1.017)	0.372	0.943(0.910–0.976)	0.001
Gravidity		1.001(0.992–1.011)	0.799	0.988(0.963–1.013)	0.341
Time from menarche to first delivery		0.956(0.933–0.978)	<0.0001	1.035(0.968–1.106)	0.313
Age at first delivery	≤23 years	1.737(1.105–2.729)	0.017	2.081(0.863–5.019)	0.103
	24–29 years	1.686(1.075–2.646)	0.023	2.124(1.096–4.113)	0.026
	≥30 years	1		1	
HT		0.524(0.407–0.675)	<0.0001	0.602(0.452–0.803)	0.001
OC		0.773(0.626–0.953)	0.016	0.635(0.497–0.812)	<0.0001

OR, odds ratio; CI, confidence interval; BMI, body mass index; HT, hormone therapy; OC, oral contraceptive.

Categorical variables were as follows: BMI (reference: over 25), smoking status (reference: non-smoker), age at first delivery (reference: ≥30 years old), HT (reference: no), OC (reference: no).

Continuous variables were as follows: age (years), calcium intake (100 mg/day), exercise (days/week), age at menarche (years), age at menopause (years), gravidity (n), time from menarche to first delivery (years).

P values were obtained using multivariate logistic regression analysis.

## Discussion

We demonstrated that a lower BMI, lower calcium intake, earlier menopause, and later menarche increase the risk of postmenopausal osteoporosis, whereas HT and OC use were associated with a decreased risk. In addition, there was a significant relationship between age at first delivery and osteoporosis, although the mean age at first delivery was comparable between the osteoporosis and non-osteoporosis groups.

In our study, a few risk factors for developing osteoporosis in postmenopausal women were expected, such as lower BMI, lower calcium intake, earlier menopause, and later menarche. Furthermore, the finding that age at first delivery was an independent risk factor for postmenopausal osteoporosis was also expected. When compared to previous studies [12,13], distinctive finding of our study was that the risk of osteoporosis is significantly increased for those who first gave birth at the age of 24–29 years, not in the adolescent period.

A few previous studies have evaluated the relationship between age at the time of first pregnancy and osteoporosis. Two studies have investigated whether pregnancy during the adolescent period may affect future bone loss [9,19]. Both studies suggested that adolescent pregnancy seems to affect neither increased bone loss or future osteoporosis. Moreover, they also suggested that pregnancy causes reversible bone loss, which is compensated for after lactation. However, it has not been established whether age at first pregnancy affects the future development of postmenopausal osteoporosis. In a cross-sectional study that was conducted using KNHANES 2008 data, a history of adolescent pregnancy seemed to significantly increase the risk of postmenopausal osteoporosis [12]. However, another cross-sectional study performed in postmenopausal women reported that there was a significantly lower incidence of osteoporosis among women who breastfed and whose first delivery occurred after 27 years of age, an age considered to be after PBM acquisition [13]. The discrepancies among studies may be explained in terms of bone mass accumulation [20]. Bone mass accumulates at a great rate during the adolescent period [21]. However, bone mass reaches its peak at a later age, namely around the late 20s [22] to early 30s [23,24]. According to the periods of bone mass acquisition, a hypothesis can be made that during rapid accumulation of bone, loss of bone mass has time to recover, considering that acquisition of bone mass resumes after pregnancy. However, towards the end of PBM accumulation, when the rate slows down, loss of bone mass at this time interrupts bone mass accumulation at a critical time. This assumption is supported by a longitudinal study conducted in 25 children with leukemia or lymphoma [25]; in this study, 8 years after allogeneic bone marrow transplant, whole-body bone mass only slightly reduced, and size-adjusted bone mass was normal. However, in current study, pregnancy at the 30s, the maintenance period of bone mass, interruption of bone mass does not seem to affect future osteoporosis risk. It may be explained by general view that pregnancy does not increase osteoporosis risk, because bone mass has been compensated after delivery [7]. The compensation after delivery may be enough for maintaining the bone mass, since it does not require increasing the bone mass, but maintaining it.

In our study, women who first delivered at the age of 24–29 years exhibited an increased risk of postmenopausal osteoporosis. In our study, PBM in the femoral neck was achieved in the early 20s and in the fourth decade for the lumbar spine (S1 Fig). These differences can be explained by ethnicity, a variable that is well known to affect PBM acquisition [26,27]. In our grouping by age at first delivery, ≤23 years was selected to represent the rapid accumulating phase of female bone mass, 24–29 years represented the slow accumulating phase before PBM acquisition, and ≥30 years represented the maintenance phase. Our results showed a significantly increased risk of postmenopausal osteoporosis in the 24–29 age group, thereby confirming this hypothesis.

In contrast with prior study results, our results indicated that exercise and current smoking status were not significantly associated with postmenopausal osteoporosis. This may be because history of smoking was not evaluated in the study. In addition, participants’ activity levels were assessed by subjective intensity of activity and did not specify the type of exercise performed. Although there is controversy regarding the relationship between age at menarche and postmenopausal osteoporosis [2,6], it seemed to have borderline significance in our study. Additionally, serum vitamin D levels were not associated with osteoporosis; this may be because there were comparable levels of vitamin D deficiency in both the osteoporosis and non-osteoporosis groups, as there is a high prevalence of vitamin D deficiency in South Korea [28].

One of the limitations of this study was the cross-sectional design. In addition, most of the data came from questionnaires based on memory recall. Although breastfeeding history is known to be a risk factor for osteoporosis, we could not include breastfeeding history or duration in the analysis because of the extent of missing data. Previous breastfeeding history and osteoporosis shall be taken into further study, although we cannot include in the current study. Since age at first delivery can be a socially conditioned factor, the impact of this factor might be complicated to interpret. For example, in the 1940s and 1950s, marriage and first delivery typically occurred during the late teens to early 20s in Korea. Therefore, adjusting for age may have eliminated the effect of early delivery (≤23 years). Similarly, there were relatively few participants whose age at first delivery was ≥30 years. Further longitudinal studies are needed to confirm the relationship between interruption in bone mass acquisition and postmenopausal osteoporosis.

In conclusion, reproductive factors, including menarche, menopause, pregnancy, and use of hormones, are significant risk factors for the development of osteoporosis in postmenopausal women. In particular, age at first delivery may affect the development of osteoporosis in the future by interrupting PBM acquisition. This emphasizes the need to consider HT use in women with the above risk factors and to address the issue of bone loss in these women with interventions such as vitamin D supplements or exercise during the antepartum to postpartum period.

## Supporting Information

S1 Table Bone mineral density and content of femoral neck, lumbar spine in total female population. (DOCX)

S1 Fig Changes in bone mineral density and content in total female population by aging. Bone mineral density (Figure A), content (Figure B) at femoral neck. Bone mineral density (Figure C), content (Figure D) at lumbar spine.
(TIF)

S1 File (DOCX)
