# Supplementary material for: Age at First Delivery and Osteoporosis Risk in Korean Postmenopausal Women: The 2008–2011 Korea National Health and Nutrition Examination Survey (KNHANES)
Source: PLoS One. 2015 May 6;10(5):e0123665. doi: 10.1371/journal.pone.0123665 (PMC4422688; doi:10.1371/journal.pone.0123665)
Supplement: S1 Table — (DOCX) [file pone.0123665.s001.docx]

**S1 table. Bone mineral density and content of femoral neck, lumbar spine in total female population.**

|  | **Femoral neck** | | | |  | **Lumbar spine, total** | | | |
| --- | --- | --- | --- | --- | --- | --- | --- | --- | --- |
|  | **BMD** | | **BMC** | |  | **BMD** | | **BMC** | |
| **AGE** | **MEAN ± SD** | **95% CI** | **MEAN ± SD** | **95% CI** | **AGE** | **MEAN ± SD** | **95% CI** | **MEAN ± SD** | **95% CI** |
| **10** | 0.604±0.081 | 0.588-0.619 | 2.522±0.485 | 2.428-2.615 | **10** | 0.637±0.095 | 0.618-0.655 | 27.302±6.235 | 26.095-28.509 |
| **11** | 0.655±0.095 | 0.637-0.673 | 2.933±0.569 | 2.826-3.04 | **11** | 0.701±0.112 | 0.68-0.722 | 33.242±8.107 | 31.717-34.767 |
| **12** | 0.714±0.113 | 0.691-0.737 | 3.302±0.6 | 3.179-3.424 | **12** | 0.788±0.118 | 0.763-0.812 | 40.396±9.077 | 38.547-42.245 |
| **13** | 0.738±0.104 | 0.719-0.756 | 3.446±0.567 | 3.344-3.549 | **13** | 0.848±0.101 | 0.829-0.866 | 45.829±8.386 | 44.313-47.345 |
| **14** | 0.739±0.112 | 0.717-0.762 | 3.514±0.613 | 3.391-3.636 | **14** | 0.871±0.1 | 0.851-0.891 | 48.08±8.046 | 46.466-49.693 |
| **15** | 0.769±0.097 | 0.747-0.79 | 3.706±0.561 | 3.582-3.829 | **15** | 0.896±0.104 | 0.873-0.919 | 51.424±8.842 | 49.481-53.367 |
| **16** | 0.782±0.109 | 0.76-0.805 | 3.725±0.586 | 3.603-3.846 | **16** | 0.922±0.119 | 0.898-0.947 | 51.712±9.083 | 49.831-53.593 |
| **17** | 0.783±0.114 | 0.759-0.807 | 3.771±0.612 | 3.641-3.9 | **17** | 0.933±0.112 | 0.91-0.957 | 54.073±9.089 | 52.147-55.998 |
| **18** | 0.77±0.094 | 0.745-0.794 | 3.679±0.525 | 3.545-3.813 | **18** | 0.924±0.105 | 0.897-0.951 | 53.518±8.699 | 51.29-55.746 |
| **19** | 0.803±0.094 | 0.786-0.821 | 3.863±0.494 | 3.77-3.955 | **19** | 0.952±0.093 | 0.934-0.969 | 55.712±7.571 | 54.295-57.129 |
| **20** | 0.78±0.095 | 0.76-0.801 | 3.765±0.552 | 3.646-3.884 | **20** | 0.934±0.109 | 0.91-0.957 | 53.763±9.202 | 51.778-55.748 |
| **21** | 0.803±0.11 | 0.782-0.824 | 3.861±0.584 | 3.749-3.973 | **21** | 0.968±0.11 | 0.946-0.989 | 56.967±9.178 | 55.207-58.725 |
| **22** | 0.79±0.107 | 0.769-0.811 | 3.781±0.55 | 3.672-3.89 | **22** | 0.958±0.107 | 0.937-0.979 | 56.033±8.218 | 54.402-57.663 |
| **23** | 0.795±0.114 | 0.775-0.815 | 3.85±0.656 | 3.733-3.968 | **23** | 0.968±0.115 | 0.947-0.989 | 57.34±9.845 | 55.575-59.104 |
| **24** | 0.767±0.102 | 0.749-0.784 | 3.766±0.57 | 3.669-3.863 | **24** | 0.956±0.113 | 0.937-0.975 | 56.89±9.181 | 55.322-58.459 |
| **25** | 0.77±0.086 | 0.754-0.787 | 3.719±0.46 | 3.63-3.808 | **25** | 0.961±0.098 | 0.943-0.980 | 57.189±8.203 | 55.579-58.738 |
| **26** | 0.764±0.103 | 0.746-0.783 | 3.705±0.525 | 3.611-3.8 | **26** | 0.964±0.106 | 0.945-0.983 | 57.14±9.087 | 55.505-58.776 |
| **27** | 0.761±0.099 | 0.744-0.778 | 3.683±0.526 | 3.595-3.771 | **27** | 0.957±0.108 | 0.939-0.975 | 57.477±8.544 | 56.05-58.905 |
| **28** | 0.739±0.084 | 0.724-0.753 | 3.588±0.47 | 3.506-3.669 | **28** | 0.953±0.11 | 0.943-0.973 | 56.396±8.302 | 54.956-57.837 |
| **29** | 0.76±0.096 | 0.744-0.775 | 3.684±0.503 | 3.601-3.766 | **29** | 0.972±0.091 | 0.957-0.987 | 58.065±7.824 | 56.785-59.345 |
| **30** | 0.753±0.094 | 0.738-0.769 | 3.647±0.507 | 3.565-3.729 | **30** | 0.973±0.113 | 0.955-0.992 | 58.174±9.404 | 56.646-59.701 |
| **31** | 0.754±0.103 | 0.739-0.769 | 3.69±0.531 | 3.611-3.769 | **31** | 0.975±0.115 | 0.958-0.992 | 59.115±9.279 | 57.73-60.499 |
| **32** | 0.766±0.11 | 0.759-0.783 | 3.744±0.589 | 3.651-3.837 | **32** | 0.996±0.116 | 0.977-1.014 | 58.946±9.216 | 57.488-60.404 |
| **33** | 0.768±0.11 | 0.752-0.784 | 3.737±0.585 | 3.652-3.822 | **33** | 0.994±0.112 | 0.978-1.011 | 59.832±9.65 | 58.428-61.235 |
| **34** | 0.755±0.099 | 0.742-0.768 | 3.684±0.529 | 3.615-3.754 | **34** | 0.995±0.116 | 0.98-1.011 | 59.447±9.209 | 58.24-60.655 |
| **35** | 0.759±0.1 | 0.759-0.756 | 3.693±0.564 | 3.622-3.764 | **35** | 0.99±0.116 | 0.975-1.004 | 59.432±9.762 | 58.208-60.655 |
| **36** | 0.757±0.104 | 0.744-0.77 | 3.685±0.535 | 3.616-3.753 | **36** | 0.997±0.106 | 0.983-1.011 | 60.168±8.921 | 59.024-61.312 |
| **37** | 0.762±0.102 | 0.749-0.776 | 3.745±0.571 | 3.667-3.822 | **37** | 1.007±0.115 | 0.992-1.023 | 60.67±9.855 | 59.333-62.008 |
| **38** | 0.755±0.108 | 0.741-0.769 | 3.693±0.57 | 3.622-3.765 | **38** | 0.999±0.123 | 0.984-1.015 | 60.123±9.917 | 58.88-61.366 |
| **39** | 0.758±0.099 | 0.745-0.771 | 3.704±0.508 | 3.637-3.77 | **39** | 1±0.114 | 0.986-1.015 | 60.547±9.277 | 59.331-61.763 |
| **40** | 0.763±0.106 | 0.749-0.777 | 3.744±0.57 | 3.669-3.819 | **40** | 1.005±0.123 | 0.989-1.021 | 60.683±10.127 | 59.349-62.016 |
| **41** | 0.753±0.102 | 0.74-0.766 | 3.697±0.591 | 3.62-3.773 | **41** | 1.001±0.119 | 0.985-1.016 | 60.606±9.653 | 59.354-61.857 |
| **42** | 0.764±0.102 | 0.749-0.779 | 3.739±0.552 | 3.66-3.818 | **42** | 0.997±0.121 | 0.979-1.014 | 60.089±10.589 | 58.578-61.6 |
| **43** | 0.769±0.095 | 0.755-0.782 | 3.783±0.526 | 3.707-3.858 | **43** | 1±0.116 | 0.983-1.016 | 59.721±9.738 | 58.323-61.118 |
| **44** | 0.775±0.101 | 0.756-0.789 | 3.8±0.549 | 3.72-3.88 | **44** | 1.002±0.119 | 0.985-1.019 | 60.413±9.379 | 59.045-61.781 |
| **45** | 0.749±0.108 | 0.733-0.764 | 3.666±0.537 | 3.589-3.744 | **45** | 0.977±0.125 | 0.959-0.995 | 57.851±9.971 | 56.405-59.298 |
| **46** | 0.754±0.104 | 0.739-0.768 | 3.748±0.552 | 3.671-3.826 | **46** | 0.992±0.117 | 0.976-1.009 | 59.841±9.502 | 58.512-61.169 |
| **47** | 0.759±0.097 | 0.746-0.771 | 3.714±0.536 | 3.643-3.785 | **47** | 0.989±0.123 | 0.972-1.005 | 58.941±9.846 | 57.635-60.247 |
| **48** | 0.756±0.107 | 0.741-0.772 | 3.725±0.548 | 3.646-3.803 | **48** | 0.981±0.126 | 0.963-0.999 | 58.49±11.11 | 56.901-60.08 |
| **49** | 0.739±0.111 | 0.725-0.754 | 3.672±0.589 | 3.593-3.751 | **49** | 0.953±0.133 | 0.935-0.971 | 56.379±10.721 | 54.944-57.813 |
| **50** | 0.727±0.101 | 0.713-0.741 | 3.617±0.571 | 3.541-3.694 | **50** | 0.932±0.140 | 0.913-0.951 | 54.876±10.584 | 53.46-56.293 |
| **51** | 0.738±0.106 | 0.724-0.751 | 3.632±0.568 | 3.559-3.705 | **51** | 0.937±0.14 | 0.919-0.955 | 55.028±11.116 | 53.594-56.46 |
| **52** | 0.711±0.103 | 0.697-0.725 | 3.498±0.553 | 3.424-3.572 | **52** | 0.911±0.128 | 0.894-0.928 | 53.223±10.383 | 51.833-54.613 |
| **53** | 0.721±0.1 | 0.708-0.734 | 3.559±0.537 | 3.488-3.629 | **53** | 0.916±0.14 | 0.897-0.934 | 52.989±11.516 | 51.48-54.499 |
| **54** | 0.698±0.101 | 0.683-0.713 | 3.437±0.509 | 3.362-3.513 | **54** | 0.884±0.134 | 0.864-0.904 | 51.207±9.689 | 49.766-52.649 |
| **55** | 0.668±0.086 | 0.655-0.681 | 3.311±0.497 | 3.237-3.385 | **55** | 0.836±0.127 | 0.819-0.857 | 47.752±9.96 | 49.266-49.238 |
| **56** | 0.675±0.102 | 0.66-0.691 | 3.321±0.528 | 3.323-3.4 | **56** | 0.837±0.121 | 0.819-0.855 | 47.495±9.595 | 46.063-48.927 |
| **57** | 0.676±0.091 | 0.662-0.691 | 3.367±0.481 | 3.291-3.444 | **57** | 0.841±0.114 | 0.823-0.859 | 48.298±9.745 | 46.751-49.844 |
| **58** | 0.669±0.092 | 0.655-0.683 | 3.312±0.506 | 3.237-3.387 | **58** | 0.836±0.136 | 0.816-0.857 | 46.265±10.473 | 44.716-47.814 |
| **59** | 0.657±0.088 | 0.644-0.671 | 3.239±0.45 | 3.171-3.308 | **59** | 0.83±0.12 | 0.812-0.848 | 47.193±9.968 | 45.671-48.716 |
| **60** | 0.638±0.093 | 0.624-0.651 | 3.156±0.509 | 3.083-3.23 | **60** | 0.813±0.122 | 0.795-0.830 | 44.759±10.634 | 43.229-46.289 |
| **61** | 0.641±0.085 | 0.628-0.654 | 3.19±0.465 | 3.118-3.261 | **61** | 0.81±0.11 | 0.793-0.827 | 44.722±9.818 | 43.203-49.24 |
| **62** | 0.64±0.091 | 0.626-0.653 | 3.178±0.515 | 3.103-3.254 | **62** | 0.814±0.123 | 0.796-0.833 | 45.377±11.692 | 43.657-47.096 |
| **63** | 0.636±0.093 | 0.622-0.649 | 3.149±0.472 | 3.08-3.218 | **63** | 0.804±0.128 | 0.786-0.823 | 44.419±10.375 | 42.897-45.941 |
| **64** | 0.622±0.08 | 0.609-0.636 | 3.074±0.442 | 2.998-3.149 | **64** | 0.779±0.116 | 0.759-0.799 | 42.54±10.398 | 40.757-44.324 |
| **65** | 0.617±0.078 | 0.603-0.63 | 3.05±0.403 | 2.98-3.119 | **65** | 0.783±0.126 | 0.761-0.804 | 41.374±11.973 | 39.32-43.428 |
| **66** | 0.604±0.081 | 0.592-0.616 | 2.996±0.446 | 2.928-3.064 | **66** | 0.773±0.125 | 0.754-0.792 | 42.093±10.689 | 40.46-43.726 |
| **67** | 0.601±0.073 | 0.591-0.612 | 2.977±0.393 | 2.92-3.034 | **67** | 0.754±0.127 | 0.735-0.772 | 40.757±11.112 | 39.15-42.365 |
| **68** | 0.592±0.084 | 0.58-0.605 | 2.941±0.453 | 2.872-3.009 | **68** | 0.764±0.115 | 0.747-0.781 | 40.407±9.919 | 38.91-41.905 |
| **69** | 0.582±0.092 | 0.569-0.596 | 2.876±0.506 | 2.801-2.95 | **69** | 0.77±0.137 | 0.75-0.79 | 40.609±11.761 | 38.884-42.334 |
| **70** | 0.575±0.086 | 0.561-0.589 | 2.827±0.424 | 2.759-2.895 | **70** | 0.749±0.124 | 0.729-0.769 | 39.442±10.533 | 37.754-41.13 |
| **71** | 0.573±0.088 | 0.559-0.587 | 2.807±0.497 | 2.729-2.884 | **71** | 0.745±0.123 | 0.726-0.764 | 37.628±10.37 | 36.004-39.252 |
| **72** | 0.565±0.081 | 0.552-0.578 | 2.751±0.456 | 2.678-2.824 | **72** | 0.743±0.133 | 0.722-0.765 | 39.02±11.285 | 37.218-40.823 |
| **73** | 0.543±0.086 | 0.528-0.557 | 2.681±0.438 | 2.605-2.757 | **73** | 0.73±0.126 | 0.708-0.752 | 36.238±10.466 | 34.422-38.055 |
| **74** | 0.551±0.093 | 0.534-0.569 | 2.738±0.829 | 2.639-2.836 | **74** | 0.758±0.141 | 0.721-0.774 | 38.074±10.752 | 36.07-40.078 |
| **75** | 0.542±0.074 | 0.527-0.556 | 2.679±0.431 | 2.594-2.764 | **75** | 0.725±0.127 | 0.7-0.75 | 37.466±11.606 | 35.186-39.745 |
| **76** | 0.52±0.082 | 0.505-0.535 | 2.557±0.419 | 2.48-2.633 | **76** | 0.717±0.131 | 0.693-0.741 | 34.788±11.169 | 32.752-36.825 |
| **77** | 0.525±0.076 | 0.51-0.541 | 2.597±0.417 | 2.513-2.681 | **77** | 0.728±0.12 | 0.704-0.752 | 34.591±10.714 | 32.443-36.739 |
| **78** | 0.512±0.088 | 0.496-0.537 | 2.565±0.477 | 2.456-2.674 | **78** | 0.726±0.141 | 0.693-0.758 | 35.588±12.582 | 32.713-38.463 |
| **79** | 0.522±0.091 | 0.498-0.545 | 2.585±0.516 | 2.453-2.717 | **79** | 0.711±0.144 | 0.675-0.748 | 36.437±12.601 | 33.21-39.664 |
| **80** | 0.492±0.082 | 0.471-0.513 | 2.49±0.446 | 2.378-2.602 | **80** | 0.697±0.135 | 0.663-0.731 | 33.286±11.679 | 30.345-36.228 |
| **81** | 0.512±0.079 | 0.49-0.533 | 2.575±0.492 | 2.44-2.709 | **81** | 0.701±0.139 | 0.663-0.739 | 35.586±11.855 | 32.35-35.822 |
| **82** | 0.471±0.085 | 0.44-0.503 | 2.348±0.369 | 2.212-2.483 | **82** | 0.673±0.111 | 0.632-0.714 | 29.887±9.255 | 26.503-33.271 |
| **83** | 0.489±0.086 | 0.459-0.518 | 2.364±0.389 | 2.23-2.497 | **83** | 0.72±0.103 | 0.684-0.755 | 35.637±9.367 | 32.42-38.855 |
| **84** | 0.446±0.076 | 0.41-0.483 | 2.204±0.367 | 2.028-2.381 | **84** | 0.675±0.125 | 0.615-0.735 | 34.909±11.145 | 29.537-40.281 |
| **85** | 0.481±0.084 | 0.442-0.520 | 2.298±0.404 | 2.109-2.488 | **85** | 0.646±0.136 | 0.582-0.709 | 32.489±9.976 | 27.82-37.158 |
