# Supplementary figures and images for: Age at First Delivery and Osteoporosis Risk in Korean Postmenopausal Women: The 2008–2011 Korea National Health and Nutrition Examination Survey (KNHANES)
Source: PLoS One. 2015 May 6;10(5):e0123665. doi: 10.1371/journal.pone.0123665 (PMC4422688; doi:10.1371/journal.pone.0123665)

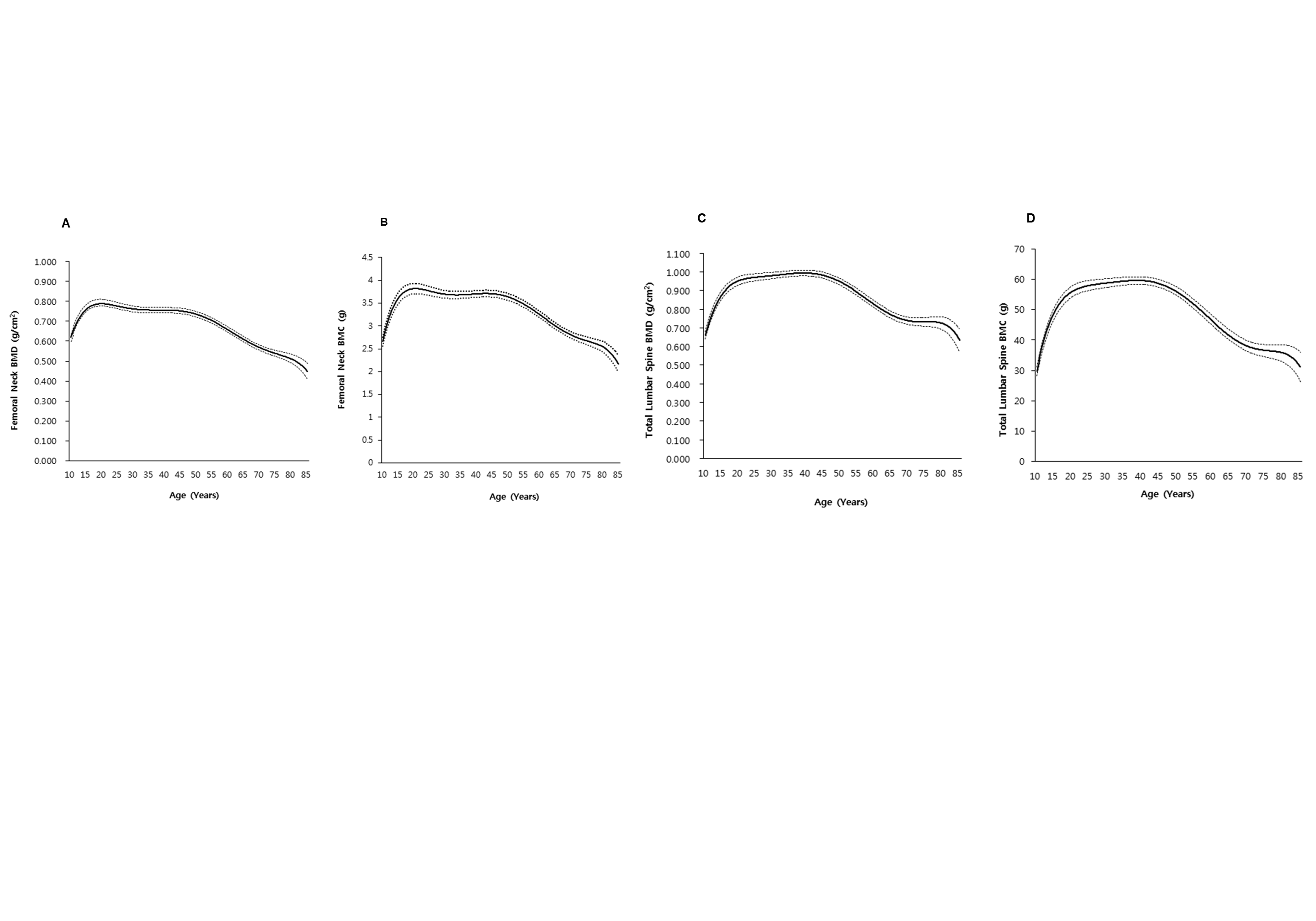

Supplement: S1 Fig — Bone mineral density (Figure A), content (Figure B) at femoral neck. Bone mineral density (Figure C), content (Figure D) at lumbar spine. (TIF) [file pone.0123665.s002.tif]
