# Supplementary material for: Age at First Delivery and Osteoporosis Risk in Korean Postmenopausal Women: The 2008–2011 Korea National Health and Nutrition Examination Survey (KNHANES)
Source: PLoS One. 2015 May 6;10(5):e0123665. doi: 10.1371/journal.pone.0123665 (PMC4422688; doi:10.1371/journal.pone.0123665)
Supplement: S1 File — (DOCX) [file pone.0123665.s003.docx]

Age at first delivery and osteoporosis risk in Korean postmenopausal women: The 2008-2011 Korea National Health and Nutrition Examination Surveys (KNHANES)

Bo Hyon Yun^1,2^, Yun Rak Choi^3^, Young Sik Choi^1,2,^ SiHyun Cho^4^, Byung Seok Lee^1,2^, Seok Kyo Seo^1,2*^

^1^Department of Obstetrics and Gynecology, Yonsei University College of Medicine, Seoul, Republic of Korea

^2^Institute of Women’s Life Medical Science, Yonsei University College of Medicine, Seoul, Republic of Korea

^3^Department of Orthopaedic Surgery, Yonsei University College of Medicine, Seoul, Republic of Korea

^4^Department of Obstetrics and Gynecology, Gangnam Severance Hospital, Yonsei University College of Medicine, Seoul, Republic of Korea

^*^Corresponding author: Seok Kyo Seo, MD

Department of Obstetrics and Gynecology, Severance Hospital, Yonsei University College of Medicine, 50 Yonsei-ro, Seodaemun-gu, 120-752, Seoul, Korea

Tel: +82-2-2228-2230; Fax: +82-2-313-8357; E-mail: tudeolseo@yuhs.ac.

**Supplementary data**

Since the time when the bone mass reaches the peak has not been established, we made a linear diagram for the bone mineral content (BMC) and bone mineral density (BMD) for our study population. Linear diagram which has shown age related changes in BMC and BMD were created including 11,860 women at 10 years old to 85 years old (S1 Fig.). Regarding the normal distribution of data and enrolled numbers of participants, we excluded women older than 85 years old. S1 Table shows the data expressed by mean±standarad deviation, 95% confidence interval.

According to our data, BMD reached at peak between 19 to 21 years old, BMC reached at peak at 19 to 23 years old at femoral neck. After reaching the peak, slightly decreased level of bone mass was shown to 35 years old, bone mass accumulation was plateaued until menopause. At lumbar spine, peak of BMD and BMC were achieved at fourth decade, most rapid accumulating rate at adolescence. Rapid accumulation of bone mass was shown until 21 to 23 years old in lumbar spine, slowing down to reach the peak gradually. We compared the accumulating velocity among ages by change of BMC or BMD per year of age, calculating the slope of the linear graphs. Therefore, we divided the age of first delivery group by 23 years, and 30 years old to show how interrupting the peak bone mass accumulation affects bone health later on. 23 years old was selected so that can stand for the rapid accumulating phase, between 23 to 30 has represented slow accumulating phase to reach the peak, and after 30 years old was divided to show the maintenance phase of the female bone mass.
